# Targeted next-generation sequencing reveals multiple deleterious variants in OPLL-associated genes

**DOI:** 10.1038/srep26962

**Published:** 2016-06-01

**Authors:** Xin Chen, Jun Guo, Tao Cai, Fengshan Zhang, Shengfa Pan, Li Zhang, Shaobo Wang, Feifei Zhou, Yinze Diao, Yanbin Zhao, Zhen Chen, Xiaoguang Liu, Zhongqiang Chen, Zhongjun Liu, Yu Sun, Jie Du

**Affiliations:** 1 Orthopaedic Department, Institute of Spinal Surgery, Peking University Third Hospital, Beijing, China; 2 Beijing Anzhen Hospital, Capital Medical University, The Key Laboratory of Remodeling-Related Cardiovascular Diseases, Ministry of Education, Beijing Collaborative Innovation Center for Cardiovascular Disorders, Beijing Institute of Heart, Lung & Blood Vessel Disease, Beijing, China

## Abstract

Ossification of the posterior longitudinal ligament of the spine (OPLL), which is characterized by ectopic bone formation in the spinal ligaments, can cause spinal-cord compression. To date, at least 11 susceptibility genes have been genetically linked to OPLL. In order to identify potential deleterious alleles in these OPLL-associated genes, we designed a capture array encompassing all coding regions of the target genes for next-generation sequencing (NGS) in a cohort of 55 unrelated patients with OPLL. By bioinformatics analyses, we successfully identified three novel and five extremely rare variants (MAF < 0.005). These variants were predicted to be deleterious by commonly used various algorithms, thereby resulting in missense mutations in four OPLL-associated genes (i.e., *COL6A1*, *COL11A2*, *FGFR1*, and *BMP2*). Furthermore, potential effects of the patient with p.Q89E of *BMP2* were confirmed by a markedly increased BMP2 level in peripheral blood samples. Notably, seven of the variants were found to be associated with the patients with continuous subtype changes by cervical spinal radiological analyses. Taken together, our findings revealed for the first time that deleterious coding variants of the four OPLL-associated genes are potentially pathogenic in the patients with OPLL.

Ossification of the posterior longitudinal ligament (OPLL, OMIM 602475) of the spine is a subset of “bone-forming” diseases, characterized by ectopic ossification in the spinal ligaments. The ossified ligament occupies the spinal canal and can eventually compress the spinal cord and nerve roots, leading to neurological disorders, such as paresthesia, paralysis and bladder bowel disturbance^1^,^2^. A mean of prevalence of OPLL was found to be 3.08% (ranging 0.44–8.92%) in Chinese populations^3^. Since the average onset age of the disease is over 50 years, OPLL is becoming a serious problem in aging societies.

The exact causes of OPLL remain elusive, although some specific genetic and environmental factors have been linked to this disease. Relative recurrence risks of up to 26.1% in parents of OPLL patients and 28.9% in siblings support the importance of genetic factors in the development of the disease^4^. Molecular genetic studies are important to the understanding of genetic etiologies of OPLL. Extensive linkage and association studies have identified multiple genes/loci that link to OPLL susceptibility, including ectonucleotide pyrophosphatase/phosphodiesterase 1 (*ENPP1*)^5^, collagen 11A2 (*COL11A2*)^6^, collagen 6A1 (*COL6A1*)^7^, bone morphogenetic protein 2 (*BMP2*)^8^, bone morphogenetic protein 4 (*BMP4*)^9^, bone morphogenetic protein *9* (*BMP9*)^10^, transforming growth factor-β1 (*TGFB1*)^11^, transforming growth factor-β3 (*TGFB3*)^12^, transforming growth factor β receptor type 2 (*TGFBR2*)^13^ and fibroblast growth factor receptor 1 (*FGFR1*)^14^ and estrogen receptor 1 (*ESR1*)^15^.

In particular, *ENPP1* was found to be a cause of a mouse model of OPLL, in addition to its association with susceptibility of OPLL^5^. Both *COL11A2*^6^ and *COL6A1*^7^ encode extracellular matrix proteins, which may provide a scaffold for osteoblastic or preosteoblastic cells or chondrocytes that subsequently proceed to membranous or endochondral ossification^16^,^17^. Bone morphogenetic protein (BMPs) (*BMP2*^8^, *BMP4*^9^ *and BMP9*^10^) were shown to induce ectopic ossification when these proteins are implanted subcutaneously^18^. Genes encoding proteins in TGF-β signaling (*TGFB1*, *TGFB3* and *TGFBR2*) are abundantly expressed in bone, and play an important role in bone metabolism. The *FGFR1* gene also plays a major role in bone development^14^. *ESR1* polymorphism was associated with the severity of OPLL only in women^15^.

By extensive linkage and association studies, at least 11 susceptibility genes/loci have been identified to be linked to OPLL susceptibility. However, all of these variants were either located in non-coding regions or were common exonic SNPs which were predicted to be non-pathogenic (Table S1). Since linkage studies either using high-density SNPs or GWAS^19^ showed limitations in pinpointing the location of the susceptibility gene, whole exome and genome sequencing are more promising approaches^20^. Also, targeted exome sequencing in a subset of disease-specific of genes has been shown, by others and us, to be a cost-efficient tool to discover novel disease-related mutations in large cohort samples^21^,^22^,^23^. By using targeted-exome sequencing, we are capable of capturing rare genetic variants that are expected to have potential damaging effects on the associated gene function.

In the present study, we performed targeted exome sequencing of the 11 OPLL-associated genes for 55 sporadic patients with OPLL, and reported the identification and characterization of potential pathogenic variants in several OPLL-associated genes. This is the first report of exonic rare variations associated with OPLL identified by targeted-exome sequencing. We also showed a possible correlation between the deleterious alleles and the severity of the OPLL phenotypes by radiological analysis of cervical spines.

## Results

### Patient Cohort

The recruited cohort consisted of 32 male and 23 female patients with an average age of 57.6 ranging from 36 to 76 years. Previous radiological analysis of OPLL in cervical spine showed four subtypes: Continuous, Segmental, Mixed and Local. Among them, the first two subtypes were found to be predominant^24^,^25^. However, radiological analysis of this cohort showed that the most predominant subtype was the Local subtype (23/55 or 41.8%), while the Continuous subtype was only found in 5 patients (9.1%). In addition, the Segmental subtype and the Mixed subtype were found in 13 patients (23.6%) and 14 patients (25.5%), respectively.

### Targeted sequencing analysis

A capture array was designed to capture all the coding sequence and intron-exon boundaries of the 11 OPLL-associated genes. By the targeted exome sequencing of the 55 samples, we obtained an average of 511.32 Mb effective sequences, and mapped a mean of 231.94 Mb to the target regions per sample. The sequencing depths on the targeted regions were yielded from 157.11 to 572.55 Mb. More than 99.2% targeted regions were covered. An average of coverage of targeted exons for >10-fold was 97.22%, and for >20-fold was 94.14% (Table S2).

### Variant identification

**Figure 1:**
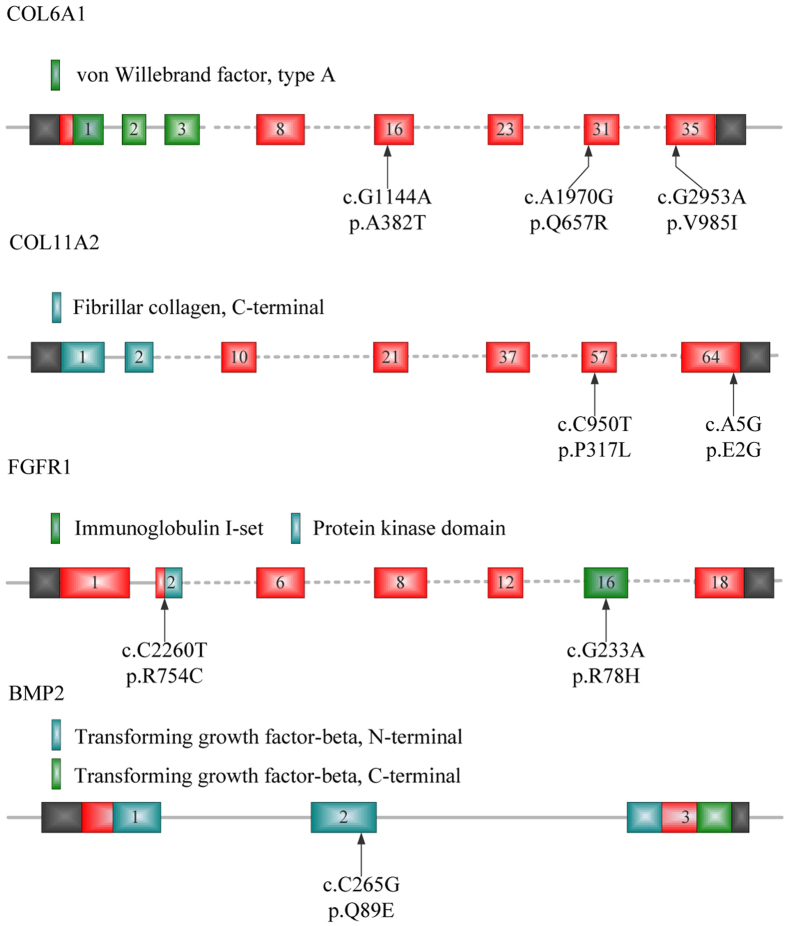
Identification of eight variants in four OPLL-associated genes. Schematic representation of variant locations. In *COL6A1*, p.A382T, p.Q657R and p.V985I are located in exon 16, exon 31, and exon 35 respectively. In *COL11A2*, p.P317L and p.E2G are in exon 57 and exon 64, respectively. In *FGFR1*, p.R78H is in exon 2 encoding a partial of kinase domain, while the p.R754C is in exon 16 encoding partial Immunoglobulin I-set motif. In *BMP2*, p.Q89E is located in transforming growth factor-beta *N*-terminal region.

**Figure 2:**
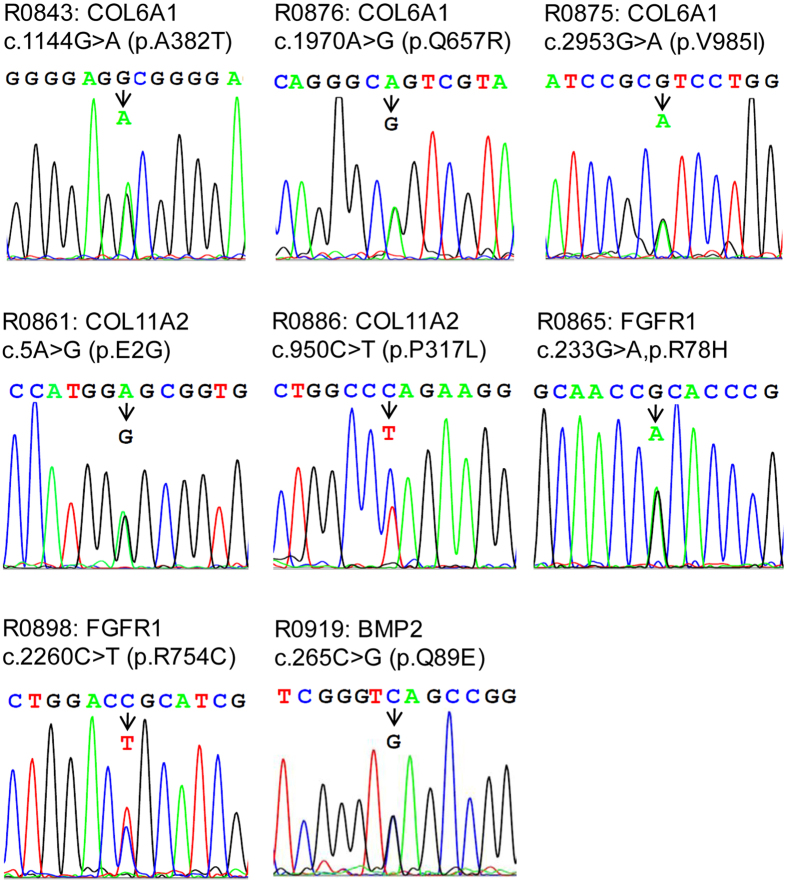
Sanger sequencing chromatograms of eight variants in four OPLL associated genes. The eight heterozygous mutations were identified in *COL6A1, COL11A2, FGFR1* and *BMP2* and are confirmed by Sanger sequencing. The ID number in each of patients is provided at the beginning of each panel.

Further bioinformatic analysis of the high-quality reads of the 55 samples identified a total of 611 variants, including 426 synonymous and 185 non-synonymous variants. In order to prioritize potential pathogenic variants, we focused on the identification of rare (MAF ≤ 0.005, based on ExAc database) and also damaging variants predicted by at least two of algorithms (i.e., SIFT, Polyphen-2, MutationTaster and GERP++). As results, eight deleterious variants or heterozygous missense mutations (Fig. 1) were identified in eight unrelated patients (Table 1), and were further confirmed by directional Sanger sequencing (Fig. 2).

**Figure 3:**
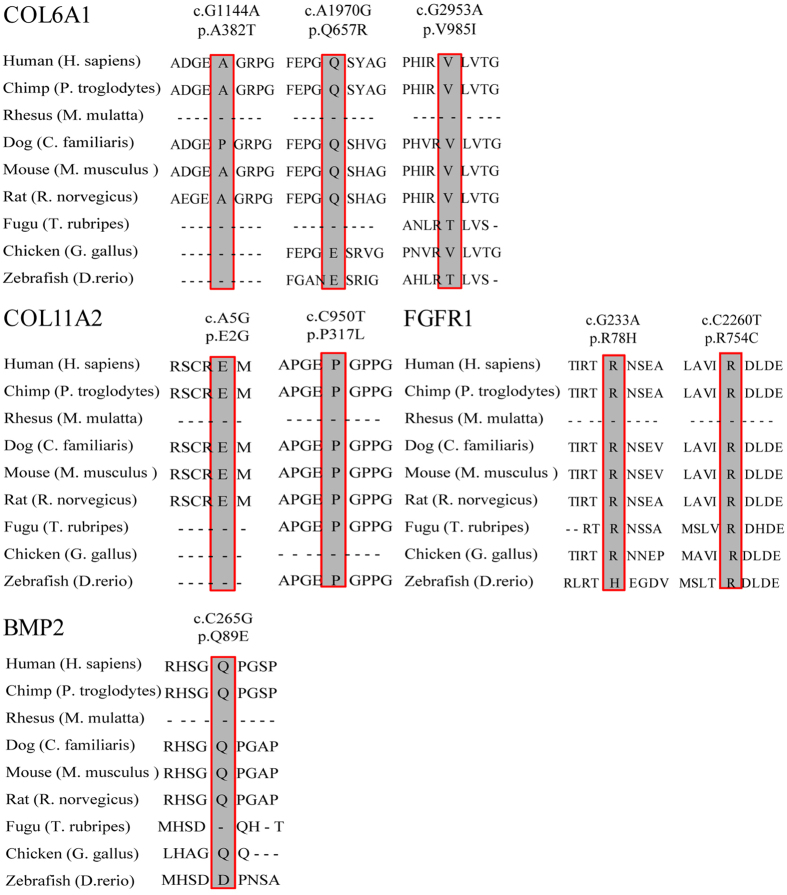
Analysis of sequence conservation in nine species of eight mutations identified in four genes associated with OPLL. All variants were subjected to the sequence conservation analysis in nine species, including Human (*H. sapiens*), Chimp (*P. troglodytes*), Rhesus (*M. mulatta*), Dog (*C. familiaris*), Mouse (*M. musculus*), Rat (*R. norvegicus*), Fugu (*T. rubripes*), Chicken (*G. gallus*), and Zebrafish (*D. rerio*). The results showed that these amino acid residues were evolutionarily conserved in vertebrates.

These variants were located in coding regions of four genes (i.e., *COL6A1*, *COL11A2*, *FGFR1* and *BMP2*), accounting for 14.5% of the OPLL patients. Three different missense mutations of *COL6A1* were found in three unrelated patients (accounting ~5.5%). The frequencies of mutations in the remaining three genes were 3.6% in *COL11A2*, 3.6% in *FGFR1*, and 1.8% in *BMP2* respectively (Table 1). The conservation of the encoded amino acid residues by these variants was analyzed in nine different vertebrate species (Fig. 3). All the eight amino acid residues as indicated were found to be evolutionarily conserved. However, no deleterious rare mutations in *ENPP1*, *BMP4*, *BMP9*, *TGFB1*, *TGFB3*, *TGFBR2* and *ESR1* were found in this cohort, suggesting highly genetic heterogeneities and complex pathogenesis.

### Analysis of BMP2 level in patient’s blood samples

To examine potential effect of the *BMP2* mutation on the BMP2 expressed cells, we measured the BMP2 level in plasma of the patient (R0919) carrying p.Q89E mutation of *BMP2* by ELISA. We found that the plasma BMP2 level (35.6 pg/ml) was significantly increased, which is about 2.1-fold higher than healthy controls (17.2 pg/ml). Furthermore, we performed RT-PCR analysis using peripheral blood cells and found that BMP2 mRNA level in the patient was approximately1.5-fold higher than the healthy controls.

### Genotype-phenotype analysis

**Figure 4:**
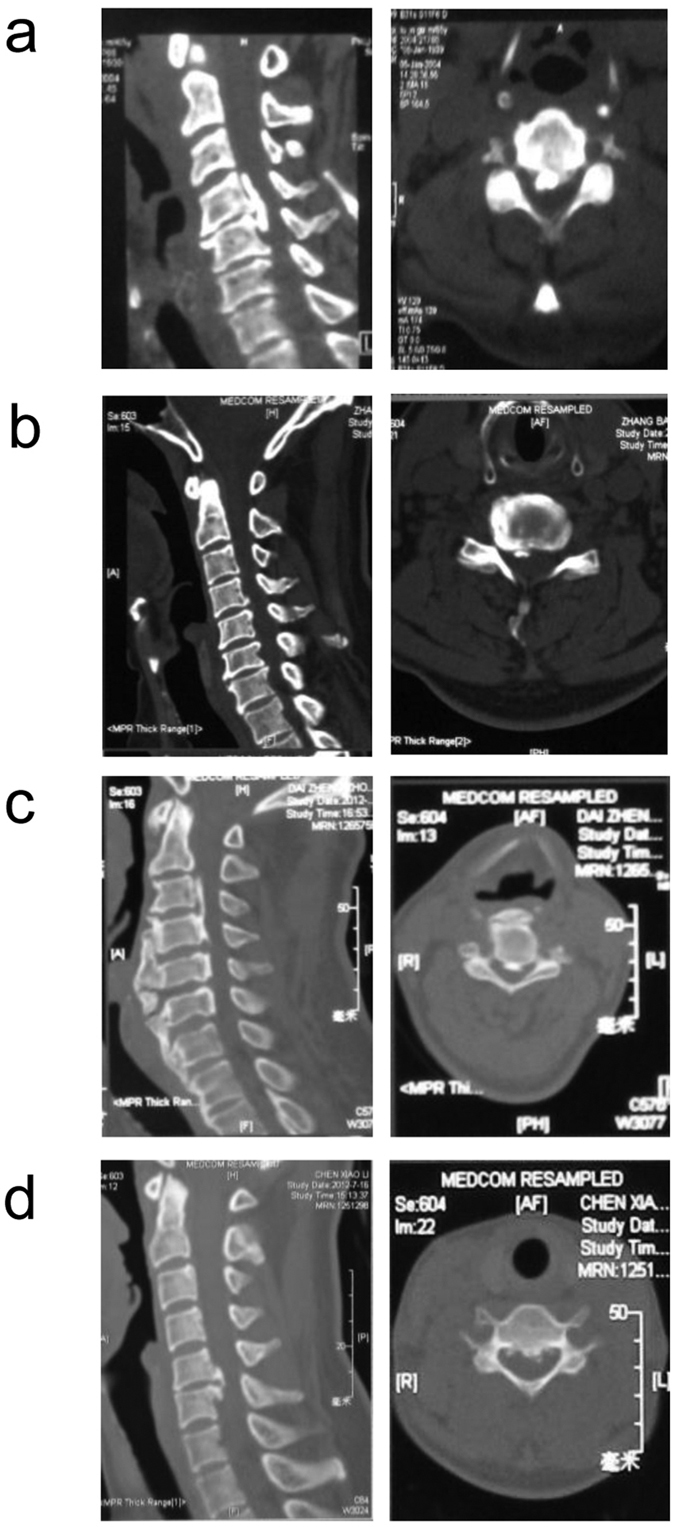
Radiological analysis of cervical spine in four subtypes of OPLL. (**a**) The Continue subtype OPLL at C4-C5 and cervical stenosis are observed in subject R0898 with c.2260C > T in *FGFR1*. (**b**) The Segmental subtype of OPLL at C4-C6 and cervical stenosis are found in subject R0865 with c.233G > A in *FGFR1*. (**c**) The Mixed subtype of OPLL at C3-C6 and cervical stenosis are seen in subject R0843 with c.1144G > A in *COL6A1*. (**d**) Also, the Local subtype of OPLL at C5 and C6 is observed in subject R0875 with c.2953G > A in *COL6A1*. Left panels: sagittal MRI; Right panels: axial CT.

A phenotype-genotype correlation was analyzed between the eight patients with missense mutations and 47 patients without significant mutations (Table 2). No difference was found between these two groups in terms of sex, age at diagnosis, and Japanese Orthopedic Association (JOA) Score. However, radiological analysis of OPLL morphology^25^ revealed that the frequency of Continuous subtype were significantly higher in patients with rare missense mutations compared to the patients without mutation (*P* = 0.018). More specifically, three mutation-positive patients (37.5%) showed the Continuous subtype (Fig. 4a), two patients (25%) showed the Segmental (Fig. 4b), two patients (25%) showed the Mixed (Fig. 4c), and only one patient (12.5%) belonged to the Local subtype (Fig. 4d). In contrast, nearly half of the mutation-negative patients (46.8%) were the Local subtype and only 4.3% of the patients without mutations were the Continuous subtype (*P* = 0.018).

## Discussion

Sufficient evidences have been shown that both genetic and environmental factors are implicated in the pathogenesis of OPLL. The feasibility of using targeted NGS technique for genetic diagnosis of inherited diseases also has been demonstrated in our previous studies^23^. In the present study, we examined all 11 reported OPLL-associated genes, and identified for the first time eight deleterious variants in four genes (*COL6A1*, *COL11A2*, *FGFR1* and *BMP2*), which significantly expanded our understanding of the mutation spectrum of OPLL and the nature of the gene function as we showed in the context.

The *COL6A1* gene encodes the α_1_ chain of the type VI collagen, an extracellular matrix protein consisting of a short central triple helix flanked by two large globular domains, in which three mutations were identified in this study. Previously, an association study with 280 OPLL patients vs. 210 controls showed that three intronic SNPs of this gene were significantly associated with OPLL. Only one of the SNPs was claimed to affect the lariat-shaped structure, thereby causing aberrant splicing^7^. Although intronic variants may attribute to the pathogenesis of the disease, their pathogenicity is very difficult to be verified. Therefore, our targeted-exome sequencing is an ideal approach to identify functional variants in exonic and exon-intron boundary regions. All of the three missense mutations of *COL6A1* identified in the present study are predicted to be deleterious by Polyphen-2 and several other algorithms, indicating adverse impacts of these amino acid substitutions on the structure and function of the encoded protein.

As a collagen family member, *COL11A2* encodes a subunit of type XI collagen, a fibril-forming minor collage, involving extracellular matrix structural functions. Previous allelic association studies between OPLL and molecular variants of the gene demonstrated that an intronic nucleotide substitution is significantly associated with OPLL and that this variant resulted in an altered splicing event, thereby possibly involving the pathogenesis of OPLL^26^. In additional studies, statistically significant associations were also established based upon several SNPs detected in the promoter, intron 6, exon 43, and exon 46. However, none of these variants were predicted to be disease-causing. Therefore, identification of harmful variants in *COL11A2* is important to link the pathogenicity of the mutated gene to OPLL^6^. As such, two different harmful mutations in exon 57 and exon 64 identified in this study provided solid evidence of the role of *COL11A2* in OPLL. Although the expression of COL11A2 protein in the nucleus pulposus of the intervertebral disc is required for maintaining its normal functions, how the mutated protein is implicated in the pathogenesis of the disease remains unclear.

It is well known that the FGF/FGFR signaling pathway plays an important role in bone development. The FGFR genes constitute a gene family of four membrane-bound receptor tyrosine kinases (*FGFR1-4*) that mediate the signals of at least 22 FGFs (*FGF1-22*). Notably, a case-control study has been previously shown that the SNP rs13317 at 3′-UTR of *FGFR1* is genetically linked to OPLL^14^. The identification of two exonic variants, in exon 2 and exon 16 respectively in our study, offered an opportunity to study the effects of these alleles on the disease in the future.

Likewise, variants in *BMP-2* has been found to be involved in different stages of development of ectopic ossification^27^, such as Ser37Ala (T/G) (i.e., rs2273073, a relatively common SNP with MAF 0.03786 in ExAc) and Ser87Ser (A/G) (i.e., rs1049007, a common SNP with MAF 0.6723 in ExAc)^8^. As shown in a recent study, a forced expression of BMP2 containing the rs2273073 in C3H10T1/2 cells (mouse embryonic fibroblasts) was found to increase cell susceptibility to bone transformation similar to pre-OPLL change as well as the sensibility to mechanical stress which might play an important role during the progression of OPLL^28^. Therefore, what we observed an increased level of *BMP2* with p.Q89E in the patient’s cells revealed its potential role in the pathogenicity of OPLL. However, further studies are required to explore how this novel missense mutation affects BMP signaling in appropriate model systems.

In summary, we have identified multiple deleterious rare variants by target NGS sequencing of 11 OPLL-associated genes in a cohort of sporadic patients with the disease, suggesting their pathogenicity in OPLL. We have also demonstrated that the combination of capture array and NGS technique is an efficient way to identify mutations related to OPLL.

## Methods

### Study participants

A total of 55 sporadic Chinese patients with TAAD were consecutively recruited between the year 2012 and 2014 from the Orthopaedic Department of Peking University Third Hospital. The research protocol was approved by the institutional ethic board. All participants provided informed consents for the clinical and genetic studies. The methods were carried out in accordance with the approved guidelines. Radiology examinations, including flexion-extension radiographs, CT scanning, and MR imaging were performed for each of the patients. The diagnosis of OPLL was made based on radiographic analyses by at least two experienced spinal surgeons. Patients with metabolic/endocrinologic disorders known to be associated with secondary OPLL, including acromegaly, hypophosphatemic rickets/osteomalacia and hyperparathyroidism, were excluded^12^. OPLL morphology was classified according to the criteria defined by the Investigation Committee on the Ossification of the Spinal Ligaments, Japanese Ministry of Public Health and Welfare. The appearance of OPLL observed in radiographs was classified into four subtypes: (1) segmental, (2) continuous, (3) mixed, and (4) local^25^. Neurological status was evaluated by using the Japanese Orthopedic Association (JOA) scoring system.

### Disease Genes Enrichment and Sequencing

DNA was extracted from whole blood with QIAamp DNA Blood kit (Qiagen, Valencia, CA, USA). Purity of the DNA samples was assessed with a NanoDrop2000 spectrophotometer (Thermo Scientific, Wilmington, DE, USA). Coding regions and exon-intron boundaries of 11 OPLL disease-associated genes, including *ENPP1* (MIM 173335), *COL11A2* (MIM 120290), *COL6A1* (MIM 120290), *BMP2* (MIM 112262), *BMP4* (MIM 112262), *BMP9* (MIM 605120), *TGFB1* (MIM 190180), *TGFB3* (MIM 190230), *TGFBR2* (MIM 190182), *FGFR1* (MIM 136350) and *ESR1* (MIM 133430) were captured by using GenCap custom enrichment kit (MyGenostics, Beijing)^29^,^30^. Biotinylated single-strand capture probes were designed to tile along the exonic non-repeated regions of the genes. The capture experiment was conducted according to manufacturer’s protocol. The enrichment libraries were sequenced on Illumina HiSeq 2000 sequencer for paired read 90-bp.

### Bioinformatics Analysis

Bioinformatics analysis began with the raw sequencing data generated from the Illumina pipeline. After adapter and low-quality reads were discarded, Burrows-Wheeler Aligner (BWA)^31^ was used to align reads to the reference sequence (Hg19). After sequences were cleaned, the final BAM files were used for variant calling. Single nucleotide polymorphisms (SNPs) were detected by SOAPsnp^32^ and small insertion/deletions (InDels) were determined by SAMtools^33^. ANNOVAR^34^ were applied to annotate the variants. Quality Control (QC) was required at each stage of the analysis pipeline.

### Validation and Evaluation of Mutations

All identified variants were bidirectionally validated by Sanger sequencing (ABI 3130XL, Applied Biosystems, Foster City, CA). Variant frequency was compared with 4 major SNP databases: ESP6500 (http://evs.gs.washington.edu/EVS/), ExAc (http://exac.broadinstitute.org/), 1000G (http://www.1000genomes.org/) and 2600 Chinese individual exomes (BGI inhouse2600) database. Potential deleterious effects of identified sequence variants were assessed by various algorithms, such as PolyPhen-2 (http://genetics.bwh.harvard.edu/pph2/)^35^, SIFT (http://sift.jcvi.org/)^36^, MutationTaster (http://www.mutationtaster.org/)^37^ as well as GERP++ (http://mendel.stanford.edu/SidowLab/downloads/gerp/)^38^.

### Protein conservative analysis

CLUSTAL W (http://www.genome.jp/tools/clustalw/) was used to compare homologous protein sequences among multiple species to analyze the consistency of amino acid sequence, especially mutations site in 9 representative species.

### Plasma collection and storage

Blood samples were collected with EDTA-containing tubes (BD, Franklin Lakes, NJ, USA); and plasma was isolated within 1 hour by centrifugation at 1900× g for 10 minutes at 4 °C to remove blood cells, and then at 16,000× g for 10 minutes at 4 °C to remove additional cellular nucleic acid attached to cell debris. Plasma samples were stored at −80 °C freezer prior to further analysis.

### Plasma BMP2 ELISA

Plasma BMP2 levels were quantified using a commercially available ELISA kit (Quantikine, BMP2 Immunoassay, R&D Systems, MN, USA). All samples were assayed according to the manufacturer’s instructions and were run in duplicate. The optical density of each well was determined using a microplate reader at 450 nm. No interference and no cross-reactivity were expected based on the manufacturer’s instructions.

### Quantitative Real-Time PCR (RT-PCR)

Total RNA was purified from blood using QIAamp RNA Blood Mini Kit (Qiagen). An on-column DNase digest (Qiagen) was performed before the clean-up step to eliminate residual genomic DNA. cDNA was synthesized from total RNA (2 μg) using reverse transcriptase M-MLV kit (Promega, Madison, WI, USA). Relative quantitative RT-PCR was applied to quantify the mRNAs levels of BMP2 and GAPDH using SYBR green Real-Time PCR master mix on the Agilent MX3000p Real-Time System (Agilent, Santa Clara, CA, USA). All experiments were performed in triplicate and normalized to GAPDH.

### Statistical analysis

Descriptive data for continuous variables are presented as mean ± SD. Student *t* test analysis was used for continuous variables. Qualitative variables were compared by Fisher test. All statistical tests were two-sided, and a *P* value of ≤0.05 was considered to be significant. SPSS (version 19, IBM, Armonk, NY, USA) was used for all statistical analyses.

## Additional Information

**How to cite this article**: Chen, X. *et al.* Targeted next-generation sequencing reveals multiple deleterious variants in OPLL-associated genes. *Sci. Rep.* **6**, 26962; doi: 10.1038/srep26962 (2016).

## Supplementary Material

Supplementary Information

## Figures and Tables

**Table 1:** Mutation and clinical finding of 55 OPLL Patients.

#	Sex	Age (y)	Gene	Nucleotide change	Protein change	ESP6500	ExAc	1000G (CHB + CHS)	BGI inhouse 2600	SIFT	PP2	Mutation Taster	GERP++	OPLL morphology	OPLL
R0843	M	61	COL6A1	c.1144G>A	p.A382T	–	0.00001655	–	–	T	**P**	**D**	S	Mixed	C3–6
R0876	M	48	COL6A1	c.1970A>G	p.Q657R	–	–	–	–	T	**P**	N	**R**	Mixed	C4–6
R0875	F	48	COL6A1	c.2953G>A	p.V985I	–	0.00001649	–	–	T	**P**	**D**	**R**	Local	C5–6
R0861	F	36	COL11A2	c.5A>G	p.E2G	–	0.00002852	0.002404	0.002404	**D**	**D**	N	S	Continuous	C5–7
R0886	M	76	COL11A2	c.950C>T	p.P317L	–	0.00028	0.002404	0.002404	**D**	**D**	**D**	**R**	Continuous	C5–7
R0865	M	64	FGFR1	c.233G>A	p.R78H	–	0.000008237	–	–	**D**	B	**D**	**R**	Segmental	C3–5
R0898	M	64	FGFR1	c.2260C>T	p.R754C	–	–	–	–	**D**	**D**	**D**	**R**	Continuous	C4–6
R0919	M	61	BMP2	c.265C>G	p.Q89E	–	–	–	–	T	B	**D**	**R**	Segmental	C5–7

Note: 1000G (CHB + CHS), 1000 Genomes (Northern Chinese + Southern Chinese); SIFT (D: deleterious, T: tolerated); PP2, Polyphen-2 (D: probably damaging, P: possibly damaging, B: benign); MutationTaster (D: disease causing, N:polymorphism); GERP++ (R: rejected_substitutions, S: substitutions).

**Table 2:** Clinical Features of Patients With or Without OPLL Gene Mutations.

Examinations	Mutation Positive (*n*= 8)	Mutation Negative (*n*= 47)	*p*
Age (yr) at diagnosis	57.38 ± 12.58	57.66 ± 9.34	NS
Number of patients at or below 52 years	3 (37.5%)	13 (27.7%)	NS
Male/female	6/2	26/21	NS
OPLL morphology by radiology analysis			0.026
Continuous	3 (37.5%)	2 (4.3%)	0.018
Segmental	2 (25%)	11 (23.4)	NS
Mixed	2 (25%)	12 (25.5%)	NS
Local	1 (12.5%)	22 (46.8%)	NS
JOA Score	14.64 ± 1.24	12.19 ± 5.09	NS

Note: Data are presented as mean ± SD or *n* (%). NS, not significant; JOA, Japanese Orthopedic Association.
