# Supplementary material for: Targeted next-generation sequencing reveals multiple deleterious variants in OPLL-associated genes
Source: Sci Rep. 2016 Jun 1;6:26962. doi: 10.1038/srep26962 (PMC4887887; doi:10.1038/srep26962)
Supplement: Supplementary Information [file srep26962-s1.pdf]

Supplementary information

Targeted next-generation sequencing reveals multiple deleterious variants in OPLL-associated genes

Xin Chen[1#], Jun Guo[2#], Tao Cai[2], Fengshan Zhang[1], Shengfa Pan[1], Li Zhang[1], Shaobo Wang[1], Feifei Zhou[1], Yinze Diao[1], Yanbin Zhao[1], Zhen Chen[1], Xiaoguang Liu[1], Zhongqiang Chen[1], Zhongjun Liu[1], Yu Sun[1*] & Jie Du[2*]

[1]Orthopaedic Department, Institute of Spinal Surgery, Peking University Third Hospital, Beijing, China,

[2]Beijing Anzhen Hospital, Capital Medical University, The Key Laboratory of Remodeling-Related Cardiovascular Diseases, Ministry of Education, Beijing Collaborative Innovation Center for Cardiovascular Disorders, Beijing Institute of Heart, Lung & Blood Vessel Disease, Beijing, China.

[#] These authors contributed equally to this work.

* Correspondence to: Yu Sun, Orthopaedic Department, Institute of Spinal Surgery, Peking University Third Hospital, No. 38 Xueyuan Road Haidian District, Beijing 100083, China. Tel: +86 10 8226 7380; Fax: +86 10 8226 7380.

Jie Du, Beijing Institute of Heart, Lung, and Blood Vessel Diseases, Beijing Anzhen Hospital Affiliated to the Capital Medical University, Beijing 100029, China. Tel.: +86 10 6445 6094; Fax: +86 10 6445 6095.

E-mail: sunyuor@vip.sina.com or jdu@bcm.edu

Supplementary tables

Supplementary Table S1 | Reported variants in genes associated with OPLL

Gene	Location	NCBI dbSNP ID	Nucleotide change	Protein change	Allele Frequency		P	Ref
					Case	Control		
COL11A2	exon 6 (+28)	-	G>A	p.Q272K	-	-	0.0012	[s1]
COL6A1	intron 32 (-29)	Non-dbSNP	T>C	-	0.335	0.216	0.000003	[s2]
COL6A1	intron 33 (+20)	rs2236486	A>G	-	0.440	0.329	0.00005	[s2]
COL6A1	intron 33 (+55)	rs2236487	A>G	-	0.439	0.327	0.00006	[s2]
COL6A1	intron 21 (+18)	rs2276254	A>C	-	0.340	0.236	0.00009	[s2]
COL6A1	exon 15 (+39)	rs1980982	T>C	Gly/Gly	0.402	0.310	0.0008	[s2]
BMP2	-	-	T>G	p.S37A	0.167	0.033	<0.001	[s3]
BMP9	intron c.347-49	rs7923671	T>C	-	0.384	0.320	0.0009	[s4]
BMP9	-	rs75024165	C>T	p.T304M	0.456	0.322	<0.001	[s4]
BMP9	-	rs34379100	A>C	-	0.420	0.323	<0.001	[s4]
TGFB3	IVS1 (-1284)	rs2268624	G>C	-	0.366	0.425	0.0004	[s5]
TGFB3	IVS1 (+3306)	rs2284792	A>G	-	0.452	0.414	0.037	[s5]
TGFBR2	intron 3 c.445-4	rs11466512	T>A	-	0.571	0.321	0.007	[s6]
TGFBR2	exon 4 c.571	rs56105708	G>A	p.V216I	0.095	0.012	0.024	[s6]
FGFR1	3'-UTR	rs13317	T>C	-	0.399	0.329	0.048	[s7]
ESR1	IVS1 (-364)	rs9340799	A>G	-	0.190	0.152	0.017	[s5]

ESR1	c.1782	rs2228480	G>A	p.T594T	0.168	0.164	0.034	[s5]

Supplementary Table S2 | Statistics for targeted next-generation sequencing

Sample	Aligned (%)	Average sequencing depth on target	Coverage of target region (%)	Fraction of target covered with at least 4X (%)	Fraction of target covered with at least 10X (%)	Fraction of target covered with at least 20X (%)
R0840	99.88	366.57	99.7	99	97.6	94.8
R0843	99.89	430.75	99.8	99.3	98	96
R0844	99.87	439.56	99.9	99.5	98.7	97.3
R0845	99.87	210.09	99.4	98.2	95.4	89.8
R0846	99.87	389.96	99.7	99.2	98.1	96.2
R0847	99.87	364.44	99.8	99.3	98	96.1
R0848	99.87	384.39	99.8	99.4	98.5	96.5
R0852	99.8	282.23	99.7	99	97.4	94.7
R0854	99.86	391.64	99.8	99.4	98.4	96.4
R0857	99.87	353.23	99.7	99.2	98.1	96
R0858	99.86	372.29	99.6	98.9	97.5	94.6
R0859	99.88	372.83	99.7	99.1	97.5	94.4
R0860	99.86	351.23	99.7	99	97.4	94.1
R0861	99.88	324.69	99.7	99	97.5	94.7

R0862	99.9	410.49	99.6	99.1	97.8	95.3
R0863	99.88	247.95	99.5	98.5	95.9	91.3
R0864	99.9	439.68	99.7	99.2	97.9	95.1
R0865	99.86	466.64	99.7	99.2	98.2	96
R0866	99.85	486.34	99.8	99.4	98.5	97
R0868	99.88	463.89	99.7	99.2	98.3	96.3
R0870	99.86	506.56	99.8	99.5	98.6	96.9
R0873	99.85	346.25	99.5	98.9	97.3	94.5
R0874	99.85	373.66	99.6	98.9	97.5	94.8
R0875	99.86	366.73	99.6	98.9	97.4	94.7
R0876	99.89	408.23	99.7	99.2	97.8	95
R0877	99.88	367.09	99.8	99.2	97.9	95.5
R0878	99.87	396.06	99.7	99.1	97.9	95.4
R0879	99.89	403.83	99.6	99	97.7	95.2
R0880	99.91	572.55	99.7	99.3	98.4	96.6
R0881	99.89	414.08	99.5	98.8	97.2	94.6
R0882	99.88	427.67	99.5	98.9	97.3	94.5
R0884	99.91	509.09	99.7	99.1	97.9	95.5
R0885	99.85	366.99	99.5	98.5	96.6	93.3
R0886	99.87	529.05	99.6	99.1	98	95.6
R0888	99.88	526.58	99.8	99.2	98	95.5
R0890	99.88	500.67	99.7	99.2	97.9	95.6

R0891	99.87	306.23	99.5	98.7	96.7	93.4
R0892	99.9	215.45	99.3	97.9	94.8	89.4
R0896	99.89	320.73	99.7	98.6	96	91.2
R0897	99.87	211.5	99.5	98.3	95.7	91
R0898	99.85	357.34	99.5	99.1	97.6	94.8
R0899	99.87	276.25	99.6	98.7	96.9	93.5
R0900	99.86	160.26	99.2	97.6	94	87.7
R0902	99.85	184.94	99.2	97.7	94.5	89.1
R0906	99.83	333.17	99.6	99	97.4	94.4
R0907	99.86	323.31	99.7	99.1	97.5	94.6
R0908	99.87	263.15	99.4	98.4	96.3	92.2
R0909	99.84	157.11	99.2	97.3	93	85.6
R0911	99.85	397.27	99.7	99	97.6	95.2
R0912	99.89	217.06	99.4	98.3	95.2	89.8
R0913	99.89	221.09	99.4	98.1	94.8	89.1
R0915	99.85	361.3	99.7	99.1	97.8	95.2
R0916	99.89	384.18	99.8	99.2	98	95.5
R0919	99.84	341.11	99.7	98.9	97.2	94.2
R0922	99.86	396.49	99.7	99.2	98.1	96.1
Average	99.87	363.49	99.6	98.9	97.2	94.1

References

s1. Maeda, S. *et al.* Gender-specific haplotype association of collagen alpha2 (XI) gene in ossification of the posterior longitudinal ligament of the spine. *J Hum Genet* **46**, 1-4 (2001).

s2. Tanaka, T. *et al.* Genomewide linkage and linkage disequilibrium analyses identify COL6A1, on chromosome 21, as the locus for ossification of the posterior longitudinal ligament of the spine. *Am J Hum Genet* **73**, 812-822 (2003).

s3. Wang, H. *et al.* Association of bone morphogenetic protein-2 gene polymorphisms with susceptibility to ossification of the posterior longitudinal ligament of the spine and its severity in Chinese patients. *Eur Spine J* **17**, 956-964 (2008).

s4. Ren, Y. *et al.* Association of a BMP9 haplotype with ossification of the posterior longitudinal ligament (OPLL) in a Chinese population. *PLoS One* **7**, e40587 (2012).

s5. Horikoshi, T. *et al.* A large-scale genetic association study of ossification of the posterior longitudinal ligament of the spine. *Hum Genet* **119**, 611-616 (2006).

s6. Jekarl, D. W. *et al.* TGFBR2 gene polymorphism is associated with ossification of the posterior longitudinal ligament. *J Clin Neurosci* **20**, 453-456 (2013).

s7. Jun, J. K. & Kim, S. M. Association study of fibroblast growth factor 2 and

fibroblast growth factor receptors gene polymorphism in korean ossification of the posterior longitudinal ligament patients. *J Korean Neurosurg Soc* **52**, 7-13 (2012).
